# Extraction and Reconstruction of Arbitrary 3D Frequency Features from the Potassium Dihydrogen Phosphate Surfaces Machined by Different Cutting Parameters

**DOI:** 10.3390/ma15217759

**Published:** 2022-11-03

**Authors:** Qilong Pang, Zihao Shu, Youlin Xu

**Affiliations:** 1 College of Mechatronics Engineering, Nanjing Forestry University, Nanjing 210037, China; 2 Jiangsu Institute of Quality and Standardization, Nanjing 210029, China

**Keywords:** machined surface, spatial frequency, cutting parameters, continuous wavelet transform, power spectrum density

## Abstract

To comprehensively analyze the effect of cutting parameters on the 3D surface topography of machined potassium dihydrogen phosphate crystals, 2D power spectrum density and continuous wavelet transform are used to extract and reconstruct the arbitrary actual 3D frequency features of machined potassium dihydrogen phosphate crystal surfaces. The 2D power spectrum density method is used to quantitatively describe the 3D surface topography of machined potassium dihydrogen phosphate crystals. The continuous wavelet transform method is applied to extract and reconstruct 3D topographies of arbitrary actual spatial frequency features in machined surfaces. The main spatial frequency features *f_x_* of the machined surfaces are 0.0056 μm^−1^, 0.0112 μm^−1^, and 0.0277 μm^−1^ with the cutting depth from 3 μm to 9 μm. With the feed rate changing from 8μm/r to 18 μm/r, the main spatial frequency features *f_x_* are 0.0056 μm^−1^–0.0277 μm^−1^. With the spindle speed from 1300 r/min to 1500 r/min, the main spatial frequency features *f_x_* are same as the main spatial frequency features of the cutting depths. The results indicate that the variation of cutting parameters affects the main spatial frequency features on the 3D surface topography. The amplitudes of the spatial middle-frequency features are increased with the increasing of cutting depth and spindle speed. The spatial low-frequency features are mainly affected via the feed rate. The spatial high-frequency features are related to the measurement noise and material properties of potassium dihydrogen phosphate. The distributional directions of the frequency features in the reconstructed 3D surface topography are consistent with the distribution directions of actual frequency features in the original surface topography. The reconstructed topographies of the spatial frequency features with maximum power spectrum density are the most similar to the original 3D surfaces. In this machining, the best 3D surface topography of the machined KDP crystals is obtained with a cutting depth *a_p_* = 3 μm, feed rate *f* = 8 μm/r and a spindle speed *n* = 1400 r/min.

## 1. Introduction

Potassium dihydrogen phosphate (KH_2_PO_4_, KDP) crystals are nonlinear, optical crystals [1] that are widely used as frequency-doubled components in high-power laser systems and inertial confinement fusion due to their superior optical performance [2]. Various factors can affect the 3D surface topography of machined KDP crystal surfaces in the machining process, such as machining conditions and tool parameters [3]. The effect of the machining process on the machined surface topography is mainly presented in the frequency features [4,5]. Some researchers have compared the effects of cutting parameters on the surface roughness of materials under dry conditions and minimum quantity lubrication [6,7]. In the process of machining, the lubrication conditions of the tool, the ambient temperature and the cutting parameters affect the machined material surface [8]. Various cooling and lubricating environments during machining have an influence on tool wear and surface roughness [9]. The level of lubrication and cooling of minimum quantity lubrication can obviously improve the machined surface texture [10]. Different cutting parameters (i.e., feed rates, cutting speeds, cutting environments) were proved to have an influence on surface aspects such as roughness, profile and topography [11]. Frequency features in the machined surface may severly affect the optical performance of the KDP crystals. The middle-frequency features on the surface of KDP crystals machined via single-point diamond flycutting will limit their ability in optical fields [12]. There are four kinds of frequency features on the surface of KDP crystals machined via micro milling, and they have different effects on the optical performance of the surface [13]. The surface scratches of KDP crystalss machined via fly-cutting have an influence on the low laser damage threshold (LDT) [14]. To analyze the effects of machined surface topography on the optical performance of KDP crystals, the 3D surface topography of actual frequency features should be precisely extracted and reconstructed from machined KDP surfaces.

Various methods and instruments were used to characterize and analyze 3D surface topographies in machined surfaces. The 3D surface characterizations of indium tin oxide and atomic force were carried out via atomic force microscopy to analyze their machined surface topography [15,16]. Portable profilometers were widely used to perform in situ inspection of surface roughness in industrial practice [17]. Multifractal (fractal measure) was used to describe the surface topography of the metal fatigue process to build a Bayesian model [18,19]. The difference method was used to extract the wear particle to create a 3D reconstruction of a wear particle surface [20]. Scratches of machined stainless steel, cemented car-bide and graphite surfaces were separated and extracted via the technique based on a morphological component analysis [21]. In the 3D reconstruction based on a laser displacement sensor, the signature method was used to determine roughness parameters of surface topography to extract the 3D point cloud objects [22]. The online reconstruction of a surface topography along the entire cutting path used a piecewise cubic Hermite algorithm to ensure original precision [23].

Power spectrum density (PSD) and wavelet methods were widely applied to analyze and evaluate frequency features of machined surfaces. A spectral analysis of single point diamond machined surfaces was carried out via PSD to analyze surface roughness [24]. The PSD method was used to analyze the surface morphology of duplex stainless steel machined via the dry and minimal quantity cooling lubricate turning process [25]. The application of the PSD method was proved to reduce the effect of frequency features measurement and data analysis errors in both detection and extraction [26]. Topography measurements at different size scales were reconstructed via PSD to quantitatively predict the functional properties of surfaces [27]. The third-order polynomial function that fits to the PSD can estimate the joint roughness coefficient value of a sample profile in the spatial frequency domain of the sample surface profile [28]. The damage-detection method based on continuous wavelet transform (CWT) was used to solve the damage detection of space structures [29]. The frequencies of grinding surface topography were divided into three frequency bands to generate the simulation topography of a grinding surface via the wavelet energy method [30]. CWT can provide information about the periods of the surface undulation and the location of these undulations in a two-dimensional (2D) domain to evaluate concrete surfaces [31]. A damage index based on 2D CWT was used to improve the accuracy of damage identification and localization for plate-like structures [32].

Most researchers obtained the topography information from machined 2D profiles to study the influence of surface frequency features on the performance of components. Compared with 3D surface topographies, 2D profiles, which are analyzed via the 1D PSD method, reflect less information of the actual frequency features. The 2D ideal profile cannot be related to the cutting parameters in practical machining. Discrete wavelet transform, which is currently the main method used to decompose frequency features, cannot obtain the information of arbitrary spatial frequency features. Differing from the studies on the 2D profiles, 3D topographies of arbitrary frequency features involved in the range of sampling are extracted and reconstructed via the combination method of 2D PSD and CWT in this paper. The results completely reveal the effect of the cutting parameters on the 3D topographies of machined KDP crystals. Furthermore, it can therefore be used to analyze the effect of 3D machined surfaces on the optical performance of KDP crystal components. All of these serve to optimize the machining parameters and achieve the desirable surface topography to enhance the optical performance of machined KDP crystals.

## 2. Materials and Methods

### 2.1. Methods

#### 2.1.1. 2D PSD Method

2D PSD can obtain all actual frequency features in the machined KDP surface. 2D PSD of 3D machined surfaces can be obtained via calculating in the orthogonal *X* and *Y* directions, respectively, and shown as [33]:

(1) $$PSD\left(f_{x},f_{y}\right)=\frac{{\left|Z\left(f_{X},f_{Y}\right)\right|}^{2}}{L^{2}}=\frac{{\left|\int \int_{-L/2}^{L/2}Z\left(x,y\right)\cdot \exp\left[-j2\pi \left(f_{X}x+f_{Y}y\right)\right]dxdy\right|}^{2}}{L_{X}L_{Y}}$$

where *Z* (*f_X_*, *f_Y_*) is the Fourier transform of *z* (*x*, *y*), *f_x_* is the spatial frequency in the *X* direction component, *f_y_* is the spatial frequency in the *Y* direction component, *L_X_* is the sampling length in the *X* direction component and *L_Y_* is the sampling length in the *Y* direction component.

#### 2.1.2. Continuous Wavelet Method

Based on the analysis of 2D PSD results, actual spatial frequency features in the machined KDP surface are extracted and reconstructed via the CWT method. CWT converts spatial point cloud data into the position-frequency space and provides spatial frequency features distribution information of amplitude at each position. 3D spatial frequency features are divided into the *X* direction and *Y* direction. In the process of decomposing 3D surface topography, after extracting spatial actual frequency features in the *X* direction, spatial actual frequency features in the *Y* direction are extracted via the CWT method.

The CWT of spatial frequency features in the *X* direction can be expressed as [34]:

(2) $$W_{\psi }f(s_{x},a)=\frac{1}{\sqrt{s_{x}}}\int_{\;-\infty }^{\;+\infty }f(x)\psi *(\frac{x-a}{s_{x}})dx$$

The CWT of spatial frequency features in the Y direction can be defined as:

(3) $$W_{\psi }f(s_{y},b)=\frac{1}{\sqrt{s_{y}}}\int_{\;-\infty }^{\;+\infty }f(y)\psi *(\frac{y-b}{s_{y}})dy$$

The Mexihat (Mexican hat in the Matlab environment) wavelet basis is isotropic and can maintain the directional characteristic of frequency features. It can also reflect the wavelength and amplitude of the spatial actual frequency features. It can be expressed as [35]:

(4) $$\varphi (x,y)=(2-x^{2}-y^{2})e^{-\frac{1}{2}(x^{2}+y^{2})}$$

After extracting the actual spatial frequency features, the extracted results are reconstructed in the *Y* direction and the *X* direction, respectively. The final reconstructed results are 3D surface topography. The scale factors in the *X* and *Y* directions are *s_x_* and *s_y_*, respectively. The relationship between *s_y_* and spatial frequency features can be expressed as:

(5) $$s_{y}=\frac{f_{c}\cdot N}{2\cdot f_{y}\cdot L_{y}\cdot \Delta }$$

where *f_c_* is the center frequency of the wavelet base of Mexihat (*f_c_* = 0.25); Δ is the measurement period of the white light interferometer; *f_y_* is the actual frequency features of the machined surface in the *Y* direction; *L_y_* is the length of the sampling boundary in the *Y* direction; *N* is the number of sampling points within the length of the sampling boundary.

The relationship between *s_x_* and spatial frequency features can be expressed as:

(6) $$s_{x}=\frac{f_{c}\cdot N}{2\cdot f_{x}\cdot L_{x}\cdot \Delta }$$

where *f_x_* is the actual frequency features of the machined surface in the *X* direction; *L_x_* is the length of the sampling boundary in the *X* direction.

The wavelet coefficients represent the similarity between the original contour signal and the wavelet basis function, which is the result of CWT. The reconstructed wavelet coefficients of spatial actual frequency features in the *Y* direction can be defined as [36]:

(7) $$w_{y}\left(t\right)=\frac{1}{C_{\psi_{y}}}\int {}_{s_{y}}\int {}_{b}\left[W_{\psi }(f)(s_{y},b)\right]\cdot \psi^{s_{y},b}(t)ds_{y}\frac{ds_{y}}{s_{y}{}^{2}}$$

where *w_y_(t)* is the reconstructed contour signal in the *Y* direction of the spatial actual frequency feature; *C_ψy_* can be expressed as:

(8) $${\;C}_{\psi_{y}}=\int_{-\infty }^{+\infty }\frac{|\psi (b){|}^{2}}{\left|b\right|}db$$

The reconstructed wavelet coefficients of spatial actual frequency features in the *X* direction can be defined as:

(9) $$w_{x}\left(t\right)=\frac{1}{C_{\psi }}\int {}_{s_{x}}\int {}_{a}\left[W_{\psi }(f)(s_{x},a)\right]\cdot \psi^{s_{x},a}(t)ds_{x}\frac{ds_{x}}{s_{x}{}^{2}}$$

where *w_x_(t)* is the reconstructed contour signal in the *X* direction of the spatial actual frequency; *C_ψx_* can be expressed as:

(10) $${\;C}_{\psi_{x}}=\int_{-\infty }^{+\infty }\frac{|\psi (a){|}^{2}}{\left|a\right|}da$$

### 2.2. Cutting Experiment

The KDP crystals were machined via single-point diamond turning. The machined surface is made up of the (001) faces of KDP crystals. The tool parameters are listed in Table 1. The 3D topographies are changed in the different machined KDP surfaces obtained via different cutting parameters. The cutting parameters in the experiments are presented in Table 2.

The 3D surface topography of machined KDP crystals is measured by a Taylor Surf CCI White Light Interferometer. The sampling area of measurement was 360 μm × 360 μm; the number of sampling points was 256 × 256 and the minimum sampling period was calculated as 1.412 μm/pixel (360 μm/255 pixels).

**Figure 1:**
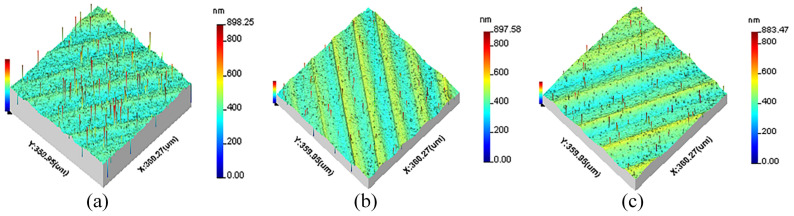
The original 3D surface topography of different cutting depths: (**a**) *a_p_* = 3 μm, (**b**) *a_p_* = 6 μm, (**c**) *a_p_* = 9 μm.

**Figure 2:**
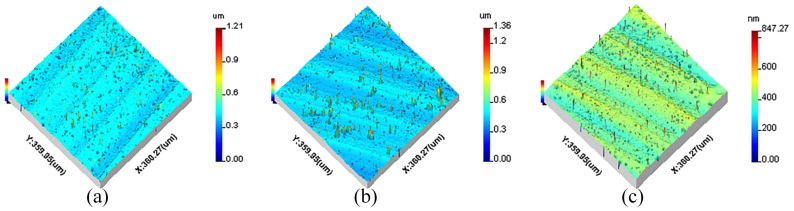
The original 3D surface topography of different feed rates: (**a**) *f* = 8 μm/r, (**b**) *f* = 12 μm/r, (**c**) *f* = 18 μm/r.

**Figure 3:**
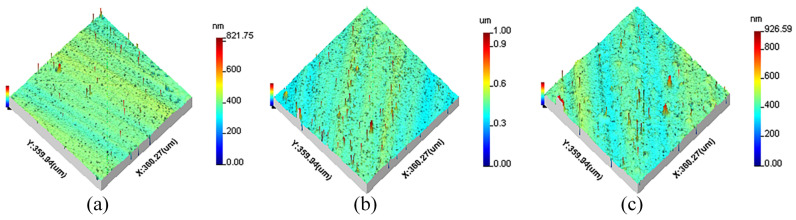
The original 3D surface topography of different spindle speeds: (**a**) *n* = 1300 r/min, (**b**) *n* = 1400 r/min, (**c**) *n* = 1500 r/min.

All measurement parameters were used in the extraction and reconstruction of 3D spatial frequencies from the machined surfaces. The 3D surface topographies of machined KDP crystals are anisotropic and shown in Figure 1, Figure 2 and Figure 3.

## 3. Extraction and Reconstruction of Frequency Features

The topographies of machined KDP crystal surfaces are composed of different 3D frequency features. Some of the frequency features are introduced via vibrations and process parameters in the machine processing and represent the low- and medium-frequency information. The others are formed via the material properties of KDP crystals and represent the high-frequency information.

### 3.1. 2D PSD Analysis

**Figure 4:**
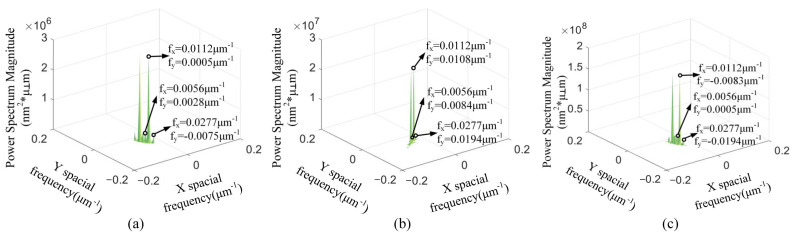
**The** 2D PSD results with different cutting depths: (**a**) *a_p_* = 3 μm, (**b**) *a_p_* = 6 μm, (**c**) *a_p_* = 9 μm.

With the cutting depth changing, Figure 4 presents that the spatial frequency with the maximum PSD values is *f_x_* = 0.0112 μm^−1^. The *f_y_* of maximum PSD values is varied in Figure 4. The spatial frequency feature *f_x_* = 0.0112 μm^−1^ is the spatial middle-frequency feature. According to Figure 4, the spatial middle-frequency features are the main spatial frequency features of the 3D machined surface topographies. The 3D machined surface topography is mainly affected via the spatial middle-frequency feature with the changing of cutting depth. The PSD values with the spatial frequency features *f_x_* = 0.0056 μm^−1^ and *f_x_* = 0.0277 μm^−1^ are smaller than the PSD with the spatial frequency features *f_x_* = 0.0112 μm^−1^. With the same spatial frequency *f_x_*, the PSD value is the smallest at the *a_p_* = 3 μm and the largest at the *a_p_* = 9 μm. This indicates that the PSD value at the same spatial frequency increases with the enlarging of the cutting depth.

**Figure 5:**
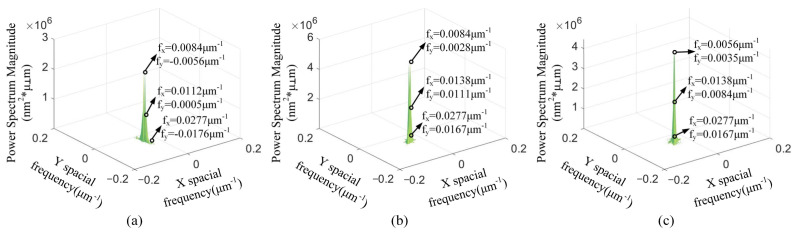
The 2D PSD results with different feed rates: (**a**) *f* = 8 μm/r, (**b**) *f* = 12 μm/r, (**c**) *f* = 18 μm/r.

With the changing of the feed rate, Figure 5 shows that the maximum PSD is at the smallest spatial frequency feature *f_x_*. When the feed rate *f* = 8 μm/r and 12 μm/r, the spatial frequency feature *f_x_* is 0.0084 μm^−1^. The spatial frequency feature *f_x_* is 0.0056 μm^−1^ at the feed rate *f* = 18 μm/r. Different from the change of the cutting depth, the spatial low-frequency feature is the main spatial frequency feature of the 3D machined surface topography. At the same spatial frequency *f_x_*, the PSD value is the smallest at the *f* = 8 μm/r and the largest at the *f* = 18 μm/r. The PSD values of the spatial low-frequency feature are enlarged with the increasing of the feed rate.

**Figure 6:**
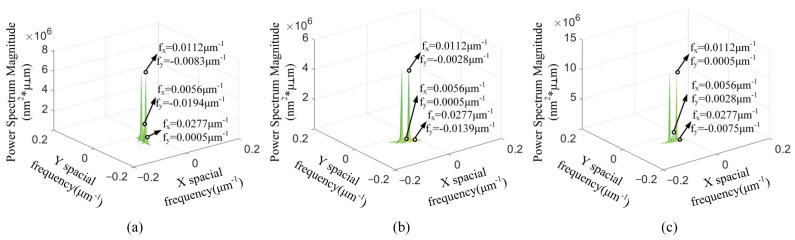
The 2D PSD results with different spindle speeds: (**a**) *n* = 1300 r/min, (**b**) *n* = 1400 r/min, (**c**) *n* = 1500 r/min.

With the variation of the spindle speed, the maximum PSD values with the spatial frequency features *f_x_* = 0.0112 μm^−1^ are shown in Figure 6. The spatial middle-frequency feature is the main spatial frequency features of the 3D machined surface topographies. The PSD values of the spatial low-frequency *f_x_* = 0.0056 μm^−1^ and the spatial high-frequency *f_x_* = 0.0277 μm^−1^ are smaller than that of the spatial middle-frequency *f_x_* = 0.0112 μm^−1^. Among these spindle speeds, the spindle speed of 1400 r/min has the smallest PSD value.

### 3.2. Reconstruction Results of Frequency Features

The actual spatial frequency features indicated in Figure 4, Figure 5 and Figure 6 are extracted and reconstructed via CWT method. The correlative calculation parameters are shown in Table 3 and Table 4. With these parameters, continuous wavelet coefficients are obtained and used to reconstruct the 3D topography of different spatial frequencies.

**Figure 7:**
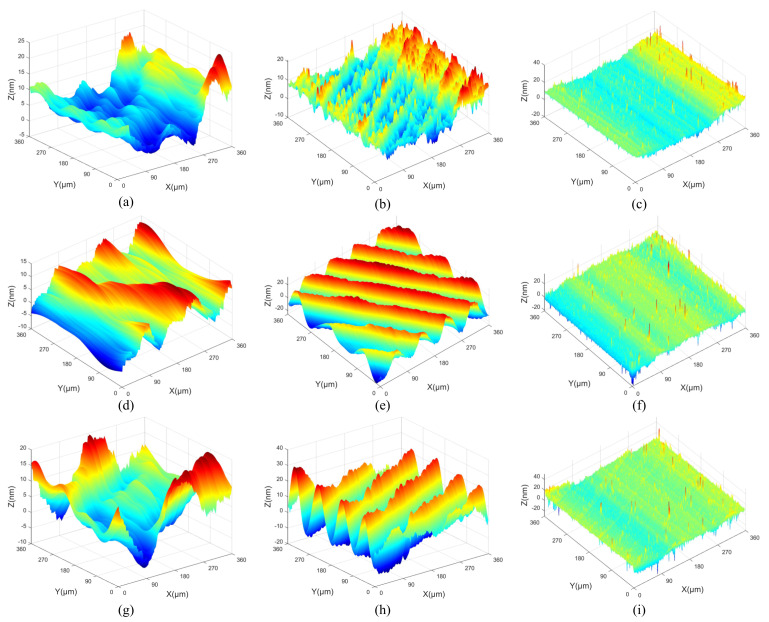
The reconstruction results of spatial frequency features with different cutting depths: (**a**) low-frequency of *a_p_* = 3 μm, (**b**) middle-frequency of *a_p_* = 3 μm, (**c**) high-frequency of *a_p_* = 3 μm, (**d**) low-frequency of *a_p_* = 6 μm, (**e**) middle-frequency of *a_p_* = 6 μm, (**f**) high-frequency of *a_p_* = 6 μm, (**g**) low-frequency of *a_p_* = 9 μm, (**h**) middle-frequency of *a_p_* = 9 μm, (**i**) high-frequency of *a_p_* = 9 μm.

Figure 7 presents the reconstructed 3D spatial frequency topographies with different cutting depths. In Figure 7a,d,g, the average amplitudes of low frequency are 5.1 nm, 8.2 nm and 9.8 nm with the cutting depth changing from 3 μm to 9 μm, and the wavelength is 178.6 μm in the X direction. The average amplitudes of middle frequency are 10 nm, 20 nm and 25 nm in Figure 7b,e,h, and the wavelength is 89.3 μm in the X direction. In Figure 7c,f,i, the average amplitudes of high-frequency are 6.9 nm, 7.6 nm and 7.4 nm, and the wavelength is 36.1 μm in the X direction. Mexihat wavelet basis maintains the directional properties of the spatial frequencies. The 3D topographies with spatial frequency *f_x_* = 0.0112 μm^−1^ (wavelength is 89.3 μm) are the most similar to the original machined surfaces and have the largest amplitudes. It can be inferred that it is the main frequency feature of the machined surfaces and the main effect factor regarding the machined surface topography. The main frequency is mainly produced via the machine processing. The 3D topography with spatial frequency *f_x_* = 0.0056 μm^−1^ (wavelength is 178.6 μm) has the maximum wavelength and median amplitude. It can be considered as the spatial low-frequency feature of the machined surface. The spatial low-frequency feature is also produced via the machine processing and can reflect the geometric error of the machined surface. The wavelengths of the main and low frequencies are invariable with the changing of cutting depth. However, the amplitudes of the main and low frequencies are increased with the enlarging of cutting depth. The 3D topography with spatial frequency *f_x_* = 0.0277 μm^−1^ (wavelength is 36.1 μm) is extremely irregular and has the minimum amplitude. It can be considered as the spatial high-frequency feature of the machined surface. The wavelengths and amplitudes of the high frequencies are almost indistinguishable with the changing of cutting depth. The reason is that the spatial high-frequency feature is unrelated to the machining process and caused via the measurement noise and material morphology of KDP crystals. As a result of reconstruction, the main and low-frequency features are strongly anisotropic surfaces, and the spatial high-frequency feature is weak anisotropy.

**Figure 8:**
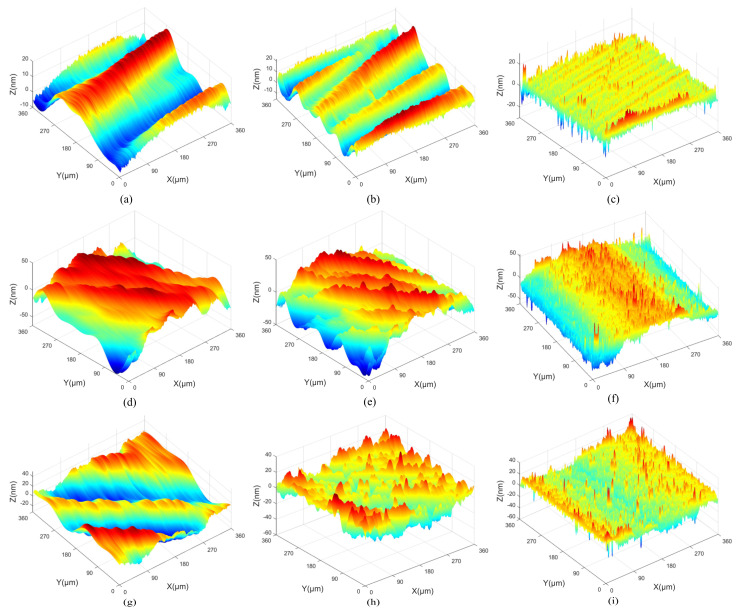
The reconstruction results of spatial frequency features with different feed rates: (**a**) low-frequency of *f* = 8 μm/r, (**b**) middle-frequency of *f* = 8 μm/r, (**c**) high-frequency of *f* = 8 μm/r, (**d**) low-frequency of *f* = 12 μm/r, (**e**) middle-frequency of *f* = 12 μm/r, (**f**) high-frequency of *f* = 12 μm/r, (**g**) low-frequency of *f* = 18 μm/r, (**h**) middle-frequency of *f* = 18 μm/r, (**i**) high-frequency of *f* = 18 μm/r.

When the feed rates are 8 μm/r, 12 μm/r and 18 μm/r, respectively, Figure 8 displays the 3D surface topographies reconstructed from the spatial frequency features obtained via CWT. In the Figure 8a,d,g, the average amplitudes of low frequency are 16.3 nm, 18.2 nm and 21.1 nm with the feed rates changing from 8 μm/r to 18 μm/r, and the wavelengths are 119 μm and 179 μm in the X direction. The average amplitudes of middle frequency are 10.1 nm, 14.9 nm and 18.9 nm in Figure 8b,e,h, and the wavelengths are 89 μm and 72 μm in the X direction. In Figure 8c,f,i, the average amplitudes of high-frequency are 5.5 nm, 8.9 nm and 10.8 nm; the wavelength is 36 μm in the X direction. The reconstructed topographies of spatial low-frequency features are the most similar to the original machined surface. Thus, the feed rate is the main factor to affect the wavelength of the low frequencies. A relatively obvious periodic change can be seen in the reconstructed 3D surface topographies of the spatial low-frequency features. Because the wavelengths of the low-frequencies are lengthened with the enlarging of the feed rates. The increasing of the feed rate can weaken the periodicity of the reconstructed 3D surface topography of the spatial low-frequency features. The results of reconstructed 3D surface topographies show that the spatial high-frequency features have short wavelengths. The 3D surface topographies of spatial high-frequency features are related to the material properties of KDP crystals. Combined with the 2D PSD results of the machined surface, the feed rate of 8 μm/r is the best machined surface.

**Figure 9:**
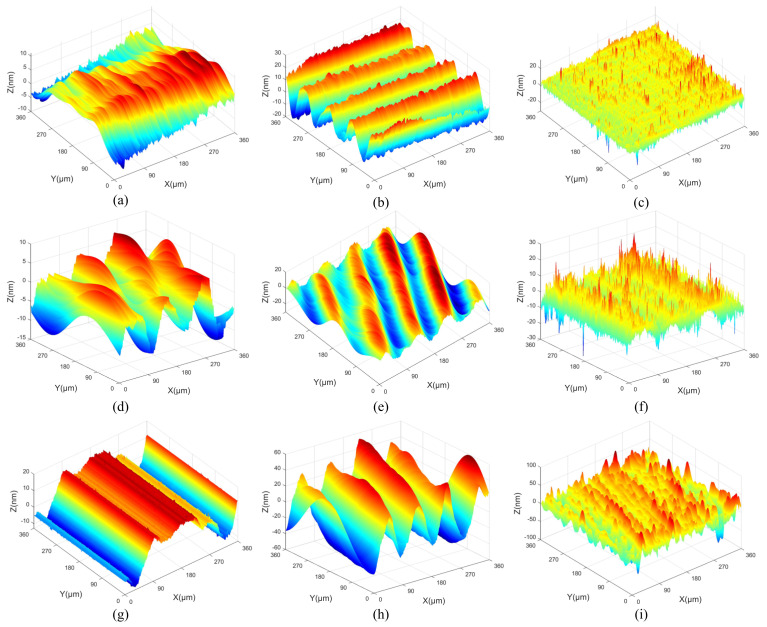
The reconstruction results of spatial frequency features with different spindle speeds: (**a**) low-frequency of *n* = 1300 r/min, (**b**) middle-frequency of *n* = 1300 r/min, (**c**) high-frequency of *n* = 1300 r/min, (**d**) low-frequency of *n* = 1400 r/min, (**e**) middle-frequency of *n* = 1400 r/min, (**f**) high-frequency of *n* = 1400 r/min, (**g**) low-frequency of *n* = 1500 r/min, (**h**) middle-frequency of *n* = 1500 r/min, (**i**) high-frequency of *n* = 1500 r/min.

Figure 9 shows the 3D surface topographies reconstructed from the spatial frequency features obtained via CWT when the spindle speeds are 1300 r/min, 1400 r/min and 1500 r/min, respectively. In Figure 9a,d,g, the average amplitudes of low frequency are 6.1 nm, 10.3 nm and 14.5 nm with the spindle speed changing from 1300 r/min to 1500 r/min, and the wavelength is 179 μm in the X direction. The average amplitudes of middle frequency are 21.8 nm, 19.8 nm and 22.5 nm in Figure 9b,e,h, and the wavelength is 89 μm in the X direction. In Figure 9c,f,i, the average amplitudes of high frequency are 5.5 nm, 8.5 nm and 10.4 nm, and the wavelength is 36 μm in the X direction. The spatial middle-frequency feature (*f_x_* = 0.0112 μm^−1^) is closest to the original machined surface. This is consistent with the analysis results showing that the spatial middle-frequency feature is the main spatial frequency feature. The wavelengths and amplitudes of the middle frequencies are almost invariable with the changing of the spindle speed. With the cutting depth and feed rate unchanged, the 3D surface topographies of the spatial low-frequency features have less information. The spatial high-frequency features reflect information about the spatial frequency features with a larger amplitude. The reconstructed 3D surface topographies of the middle and low frequency features with the spindle speed *n* = 1400 r/min are relatively flat. Combined with the 2D PSD results of the machined surface, the machined surface with *n* = 1400 r/min has the better surface quality.

### 3.3. 2D PSD of Reconstructed Topographies

**Figure 10:**
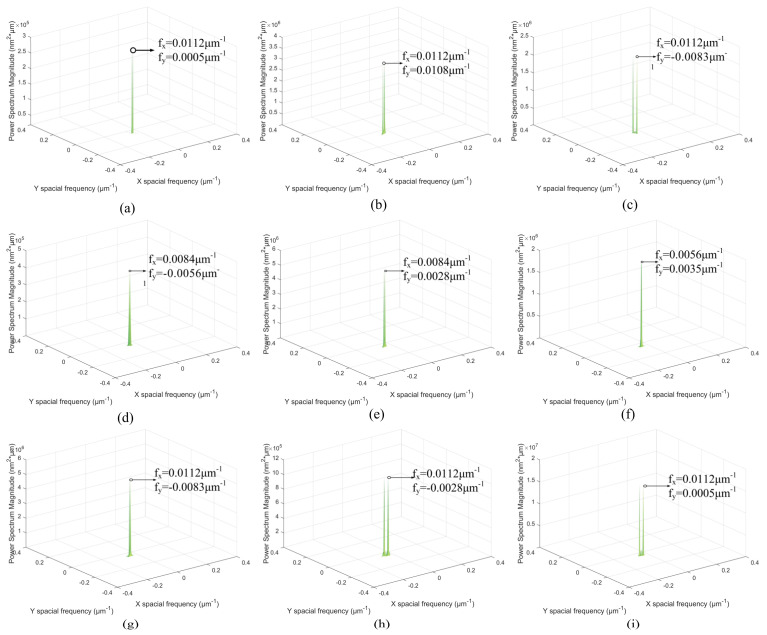
The 2D PSD results of reconstructed frequency features: (**a**) middle-frequency of *a_p_* = 3 μm, (**b**) middle-frequency of *a_p_* = 6 μm, (**c**) middle-frequency of *a_p_* = 9 μm, (**d**) low-frequency of *f* = 8 μm/r, (**e**) low-frequency of *f* = 12 μm/r, (**f**) low-frequency of *f* = 18 μm/r, (**g**) middle-frequency of *n* = 1300 r/min, (**h**) middle-frequency of *n* = 1400 r/min, (**i**) middle-frequency of *n* = 1500 r/min.

The reconstructed 3D surface topographies of the main spatial frequency features are analyzed via 2D PSD and the results are shown in Figure 10. When the cutting depth or spindle speed are of individual variation, the spatial middle-frequency features do not involve any other spatial frequency features. These spatial frequency features are consistent with the directional formation of the originally machined surface. When the feed rate changes, the spatial low-frequency frequency features are the main spatial frequency on the 3D surface topographies and their wavelengths are enlarged with the increasing of the feed rate.

In this paper, based on the analysis results of 2D PSD and CWT, the best 3D surface topography of the machined KDP crystals is obtained with a cutting depth *a_p_* = 3 μm, feed rate *f* = 8 μm/r and a spindle speed *n* = 1400 r/min because its main frequency feature is the flattest and has the best optical performance.

## 4. Conclusions

The results of our analysis lead to the following conclusions:

(1). The combination of 2D PSD and CWT is a general method to analyze the actual 3D frequency features from the arbitrary 3D original surfaces or time series signal in the machining process. The 2D PSD method can quantitatively distinguish all frequencies involved in the sampling area. The CWT method can extract and reconstruct arbitrary spatial frequency features existing in the machined surfaces.
(2). The variation of cutting parameters affects the main spatial frequency features on the 3D surface topography. The cutting depth and spindle speed mainly affect the spatial middle-frequency features. The spatial low-frequency features are mainly affected via the feed rate. The spatial high-frequency features are related to the measurement noise and crystal properties.
(3). The main frequencies in the KDP surfaces machined via different cutting depths are middle-frequency. The cutting depths cannot change the wavelengths of the main frequencies, but do have an impact on the amplitudes. With the enlarging of cutting depth, the amplitude of middle-frequency is increased.
(4). The feed rate is the main factor affecting the low frequencies of the machined KDP crystals. With the increasing of the feed rate, the wavelength and amplitude of the low frequency are obviously enlarged, but the amplification of the amplitude is less than that of the wavelength.
(5). The spindle speed cannot change the wavelength and amplitude of the middle-frequency in the machined surface. The wavelength of the low frequency is also invariable with the changing of the spindle speed. However, the amplitude of the low frequency is amplified with the increasing of the spindle speed.
(6). The directional property of spatial frequency is completely retained with Mexihat wavelet basis. The spatial low-frequency and middle-frequency features are formed in the machining process. Their distribution direction is consistent with the originally machined surface topography. The spatial high-frequency features are composed of many frequency features with short wavelengths and small amplitudes, and it is unrelated to the machining process, having no obvious directivity.
(7). The spatial low frequency and middle frequency mainly distribute in the direction perpendicular to the cutting speed. Resulting from the 3D frequency topographies, the frequencies along the direction of the cutting speed have almost no effect on the 3D topographies of the original machined surfaces.

## Figures and Tables

**Table 1:** The parameters used in SPDT to machine the KDP.

Diamond Turning Tool Nose Radius *r* (mm)	Rake Angle *γ*_0_ (°)	Clearance Angle *α*_0_ (°)
3.2	0	9

**Table 2:** The different machining parameters.

	Cutting Depth *a_p_*/μm	Spindle Speed *n*/(r/min)	Feed Rate *f*/(μm/r)
1	3	1400	12
2	6	1400	12
3	9	1400	12
4	3	1300	12
5	3	1400	12
6	3	1500	12
7	3	1300	8
8	3	1300	12
9	3	1300	18

**Table 3:** CWT calculation parameters of the spatial frequency features in the X direction.

Spatial Frequency Features/*f(*μm^−1^)	Scale/*s_x_*
0.0056	11.2415
0.0084	7.4943
0.0112	5.6208
0.0138	4.5618
0.0277	2.2727

**Table 4:** CWT calculation parameters of the spatial frequency features in the Y direction.

Spatial Frequency Features/*f*(μm^−1^)	Scale/*s_y_*
0.0005	125.9049
0.0028	22.4830
0.0035	17.9864
0.0056	11.2415
0.0075	8.3937
0.0083	7.5846
0.0084	7.4943
0.0108	5.8289
0.0111	5.6714
0.0139	4.5290
0.0167	3.7696
0.0176	3.5768
0.0194	3.2449

## Data Availability

Not applicable.
